# EBV infection of primary colonic epithelial cells causes inflammation, DDR and autophagy dysregulation, effects that may predispose to IBD and carcinogenesis

**DOI:** 10.1016/j.virusres.2023.199236

**Published:** 2023-10-12

**Authors:** Roberta Santarelli, Lorenzo Evangelista, Chiara Pompili, Salvatore Lo Presti, Alberto Rossi, Andrea Arena, Aurelia Gaeta, Roberta Gonnella, Maria Saveria Gilardini Montani, Mara Cirone

**Affiliations:** a Department of Experimental Medicine, “Sapienza” University of Rome, 00161 Rome, Italy; b Department of Molecular Medicine, "Sapienza" University of Rome, 00161 Rome, Italy

**Keywords:** EBV, IBD, Colon cancer, Cytokines, ERK1–2, Methylation

## Abstract

EBV is a gammaherpesvirus strongly associated to human cancer. The virus has been shown to play a role also in inflammatory diseases, including IBD, in the context of which colon cancer more frequently arise. In this study, we show for the first time that EBV infects primary colonic epithelial cells (HCoEpC), promotes pro-inflammatory cytokine secretion and activates molecular pathways bridging inflammation and cancer, such as ERK1/2. These effects, occurring in the course of the lytic phase of the viral life cycle, led to DDR and autophagy dysregulation. Such cellular responses, playing a key role in the maintenance of proteostasis and genome integrity, are essential to prevent carcinogenesis. Interestingly, we found that the use of the demethylating agent 5-AZA could counteract most of the effects induced by EBV infection in HCoEpC, suggesting that DNA hyper-methylation may strongly contribute to viral-driven inflammation and colon cancer predisposition.

## Highlights

• EBV infects primary colonic epithelial cells (HCoEpC) leading to the expression of latent and lytic antigens and viral release.
• EBV induces ERK1/2 activation and increases inflammatory cytokine and chemokine secretion by HCoEpC.
• EBV impairs DDR and autophagy in HCoEpC in correlation with ERK1/2 activation, effects counteracted by the demethylating agent 5-AZA.

## Graphical abstract

**Figure:**
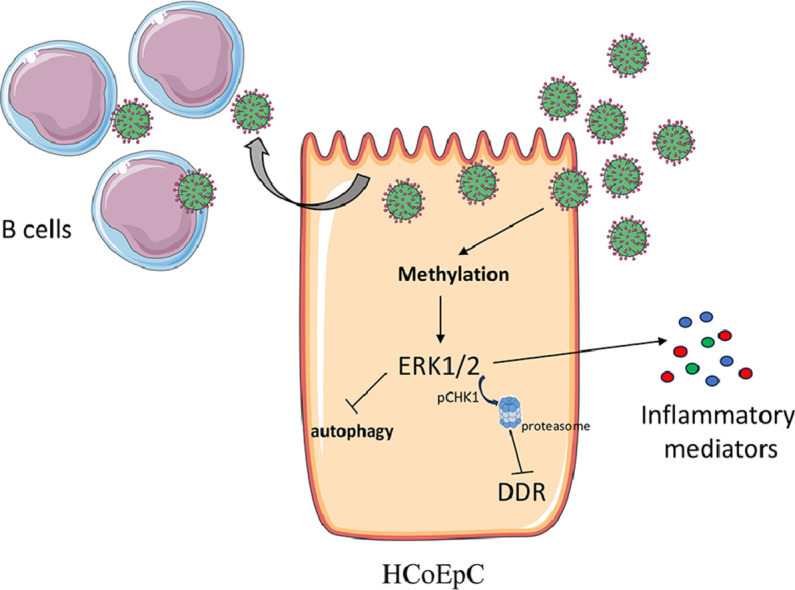

## 1 Introduction

Epstein–Barr Virus (EBV) has been the first human oncovirus to be discovered and several lymphomas and carcinomas have been associated with it over the time (Ko, 2015). These cancers arise from the main targets of EBV infection, represented by B lymphocytes and epithelial cells. The viral proteins expressed during the latent infection have been considered the most leading to oncogenic transformation (El-Sharkawy et al., 2018; Gaur et al., 2015). However, the contribution of early and late lytic antigens to viral carcinogenesis has been also acknowledged, both for EBV-associated lymphomas and for carcinomas, such as nasopharyngeal (NPC) and gastric carcinomas (GC) (Rosemarie and Sugden, 2020; Fang et al., 2012). This is not surprising, since cells permissive for a complete EBV lytic cycle and eventually dying may represent only a small minority of infected cells and that the viral particles released can activate uninfected bystander neighbor cells. Moreover, replication may spread the infection and increase the number of highly proliferating cells that for this reason more easily undergo DNA mutations. More importantly, it has been reported that, during the course of EBV lytic cycle, a higher amount of pro-inflammatory cytokines can be released (Rosemarie and Sugden, 2020), effect that may increase the risk of cancer onset. Moreover, chronic inflammation is accompanied by immune dysfunction, which may further predispose to carcinogenesis, particularly in the case of cancers associated with microbial infections (Greten and Grivennikov, 2019). The clinical examples highlighting the inter-connection between chronic inflammation and cancers are numerous and among those, the increased risk of colon cancer in patients suffering of Inflammatory Bowel Diseases (IBD) represents one of the clearest (Axelrad et al., 2016). IBD, which includes Ulcerative colitis (UC) and Crohn's disease (CD), afflict a high percentage of adult population with an incidence that is growing in industrialized countries. Interestingly, the most severe forms of IBD (Xu et al., 2020) and those refractory to therapy (Ciccocioppo et al., 2016) have been associated with EBV infection, suggesting that a possible role of EBV in the pathogenesis of these diseases deserves further investigations. The activation of molecular pathways, such as extracellular signal-regulated kinase 1/2 (ERK1/2), triggered also by pro-inflammatory cytokines, frequently occurs in the context of IBD (Broom et al., 2009) as well as in colon cancer (Tan et al., 2020) and it has been reported to be activate by EBV (Lee et al., 2008; Ma et al., 2018). Besides being associated with the undifferentiated forms of NPC and with 10% of GC cases, EBV is currently debated as a potential cofactor increasing the risk of colon carcinogenesis (Bedri et al., 2019). The virus could favor this process by promoting inflammation and its-interconnected activation of pro-oncogenic pathways in colonic cells, as it does in other cell types (Luo et al., 2021; Granato et al., 2019).

Indeed, chronic inflammation may predispose to DNA damage (Nakad and Schumacher, 2016), which represents an important mechanism linking chronic inflammation to carcinogenesis. Notably, chronic inflammation (Wielscher et al., 2022), IBD (Low et al., 2013) and cancer (Kulis and Esteller, 2010) have been correlated with DNA hypermethylation. This epigenetic modification, catalyzed by DNA methyltransferases, may deregulate gene expression, particularly that of onco-suppressors and oncogenes (Lakshminarasimhan and Liang, 2016). An increased methylation has been observed in colitis-associated colon cancer (Hartnett and Egan, 2012) and in general in gastrointestinal cancers more often than in other cancer types (Karatzas et al., 2014). Methylation may affect the activity of DNA damage response (DDR) gene promoters (Mijnes et al., 2018), impairing DNA damage repair, event observed in the context of inflammation and cancer (Christmann and Kaina, 2019; Blanco et al., 2007).

ERK1/2, whose activation can be influenced by methylation (Guo et al., 2017), may, among other processes, affect DDR (Peixoto et al., 2019), being required to re-initiate cell proliferation after DNA damage (Razidlo et al., 2009). Methylation may influence another response strongly regulating carcinogenesis, namely autophagy. Indeed, autophagy-related gene (ATG) promoter methylation may negatively regulate the autophagic process (Peixoto et al., 2019). Interestingly, autophagy deficiency, which may occur in correlation with ERK1/2 activation, has been reported to be involved in colitis (Kubota et al., 2019) and to play a key role in cancer onset (Cirone et al., 2019). EBV is able to block the autophagic process during the replicative life cycle (Granato et al., 2014) and alter DDR, effects contributing to viral replication and carcinogenesis (Hau and Tsao, 2017; Gilardini Montani et al., 2022). However, although EBV is also known to induce host gene methylation as a pro-tumorigenic mechanism (Zhang et al., 2022), whether DNA methylation may influence ERK1/2 activation, DDR and autophagy remains to be explored, particularly in the context of virus-infected colonic cells. Based on this background, in the present study, we investigated whether EBV could infect primary human colonic epithelial cells (HCoEpC) and if latent and lytic infection could influence pro-inflammatory cytokine release, the activation of pathways such as ERK1/2 and affect processes involved in carcinogenesis as DDR and autophagy. We also explored whether DNA methylation could contribute to dysregulate these processes, and at this aim, we exposed the EBV-infected HCoEpC to DNA de-methylating agent 5-azacytidine (5-AZA).

## 2 Materials and methods

### 2.1 Cell cultures

Human colonic epithelial cells (HCoEpC; iXCells Biotechnologies) were cultured in Epithelial Cell Growth Medium (iXCells Biotechnologies, Cat# MD-0041) and all experiments were performed by seeding 5 × 10^4^ HCoEpC /well in 6-well plates. B95–8, an EBV positive marmoset cell line, was cultured in RPMI 1640 (Sigma-Aldrich, St Louis, MO, USA, R0883), 10% fetal bovine serum (FBS; Sigma-Aldrich, F7524), 2 mM glutamine (Aurogene, Rome, Italy, AU-X0550), 100 mg/ml streptomycin and 100 U/ml penicillin (Aurogene, AU-L0022), in 5% CO_2_-saturated humidity at 37 °C. All experiments were performed with mycoplasma-free cells.

Human peripheral blood mononuclear cells (PBMCs) were obtained from buffy coats of healthy donors through lymphocyte cell separation medium (Cedarlane, CL5020) and B lymphocytes were isolated by immunomagnetic cell separation using anti-CD19-conjugated microbeads, according to the manufacturer's instructions (Miltenyi Biotec, 130–050–301). B cells were cultured in complete medium, in 5% CO_2_-saturated humidity at 37 °C.

### 2.2 EBV isolation and HCOEPC infection

In order to induce EBV production, B95–8 cells were treated with 12-O-tetradecanoylforbol-13-acetate (TPA, 30 ng/ml, Sigma-Aldrich; P8139) and Sodium-butyrate (3 mM, Sigma-Aldrich; B5887) for 96 h. Cells were then centrifuged at 1500 rpm for 5 min at 4 °C and the supernatant was filtered through 0.45 μm pore-size filters. Next, the virus was collected by ultracentrifugation at 29.000 rpm for 90 min at 4 °C. Pellets containing EBV were resuspended in Epithelial Cell Growth Medium and viral copy number was estimated by qPCR using ELITE MGB kit (ELITech). Subsequently, virus was aliquoted (10^7^ EBV DNA copies/50 μl) and stored at −80 °C.

Next, 5 × 10^4^ HCoEpC /well were seeded in six-well plate, grown to 80% confluence, and exposed to 10^7^ EBV DNA copies or mock-infected, in a final volume of 500μl, for one hour in incubator. Next, after adding 1,5 ml of Epithelial Cell Growth Medium, cells were spinoculated at 1400 rpm for a hour at 37 °C. Subsequently, the supernatant was removed and 2 ml of fresh Epithelial Cell Growth Medium were added. Cells were then grown at 37 °C and analyzed at different time point post-infection (p.i.).

### 2.3 Real-time quantitative polymerase chain reaction (qPCR)

To assess HCoEpC infection, cells were first washed with phosphate-buffered saline (PBS; Corning) and then treated with 0.25% trypsin-EDTA for 30 min as well as with 1 u/μl DNaseI (Norgen Biotek Corp., 25,710) for additional 30 min at 37 °C, to remove the non-internalized virus and extracellular viral DNA, respectively. Subsequently, qPCR was performed to detect and quantify the intracellular EBV DNA using ELITE MGB kit (ELITech). Mock-infected HCoEpC were used as negative control.

Furthermore, to evaluate whether the infected cells could release viral progeny, qPCR was performed on mock- and EBV-infected HCoEpC supernatants, treated with 1 u/μl of DNaseI for 30 min at 37 °C.

### 2.4 RNA isolation, reverse transcription and quantitative real time polymerase chain reaction (qRT-PCR)

qRT-PCR analyses were performed to analyze EBV lytic and latent gene expression. RNA was isolated from mock- and EBV-infected HCoEpC by using TRIzol™ Reagent (Life Technologies Corporation, Carlsbad, CA, USA, 15,596,026), according to the manufacturer's instructions. Purified RNA was treated with RNase-free DNase I (Norgen Biotek Corp.), for 10 min at RT, to remove DNA contamination. Next, reverse transcription was carried out by using High-Capacity cDNA Reverse Transcription kit (Applied Biosystems, 4,368,814) and Real Time-PCR with SensiFast SYBR No-ROX kit (Bioline).

Primers used:

LMP1 Fw-5′- AATTTGCACGGACAGGCATT −3′
LMP1 Rv-5′- AAGGCCAAAAGCTGCCAGAT −3′
BZLF1 Fw-5′-TCGCATTCCTCCAGCGATT-3′;
BZLF1 Rv-5′-CAAGGACAACAGCTAGCAGACATT-3′
gp220 Fw-5′-CCTGTGTTATATTTTCACCACTTTC-3′
gp220 Rv-5′-ACCGCACCTGCAAGCA-3′
Actin Fw-5′-TCATGAAGTGTGACGTGGACATC-3′
Actin Rv-5′-CAGGAGGAGCAATGATCTTGATCT-3′
EBNA1 Fw-5′- 5′-TCGGCTTCTGGCGTGTGACC-3′
EBNA1 Rv-5′- 5′-CATAGCGTAAAAGGAGCAACA-3′

### 2.5 Cell viability assay

Mock- or EBV-infected HCoEpC viability was determined by using a cell proliferation kit (MTT, Roche) and an Absorbance 96 reader (Byonoy GmbH, Hamburg, Germany) at different days p.i., according to the manufacturer's instructions, as previously described (Santarelli et al., 2022).

### 2.6 Indirect immunofluorescence assay (IFA)

2 × 10^4^ HCoEpC were seeded on round cover glasses in 24-well plate in a final volume of 500 μl. Upon reaching 70% confluency, cells were exposed to 2 × 10^6^ EBV DNA copies or were mock-infected, in a final volume of 100 μl, for one hour in incubator. Next, after adding 400 μl of Epithelial Cell Growth Medium, cells were spinoculated at 1400 rpm for a further hour at 37 °C. Then, the supernatant was removed and 500 μl of fresh Epithelial Cell Growth Medium were added. HCoEpC were then grown at 37 °C for 15 days and, subsequently, were washed in PBS and fixed in Paraformaldehyde (2%, Electron Microscopy Sciences, Hatfield, PA, USA) for 30 min. Cells were then washed in PBS and permeabilized with Triton-X 100 0.15% for 5 min. After three washes in PBS, HCoEpC were incubated with BSA 3%-Glycine 1% for 30 min and then with mouse monoclonal anti-ZEBRA (BZ1) (1:100 Santa CruzBiotechnology Inc., Cat# sc-53,904, Dallas, TX, USA) for 1 h at room temperature. Next, cells were washed thoroughly in PBS and then further incubated with Cy3-conjugated affiniPure sheep anti-mouse (Jackson Immuno Research Labs, Cat# 515–165–003; 1:1000, West Grove, PA, USA) for additional 30 min. Finally, cells were washed in PBS and nuclei were stained with 4′,6-diamidino-2-phenylindole (DAPI) for 1 min. After three washes with PBS, cover glasses were mounted with PBS:Glycerol (1:1) onto microscope slides and analysis was performed with Olympus BX53 (Shinjuku-ku, Tokyo, Japan) fluorescence microscope at 20 × magnification.

### 2.7 Reagents and treatments

Depending on the experiment, HCoEpC were pre-incubated for 45 min with ERK1/2 inhibitor PD0325901 (1 μM, PD, MedChemExpress, HY-10,254) or with herpesvirus DNA replication inhibitor Phosphonacetic acid (500 μM, PAA, Sigma-Aldrich, 284,270) or with the DNA demethylating agent 5-Azacytidine (0.5 μM, 5-Aza, MedChemExpress, 10,586) and then exposed to EBV as described above.

To evaluate the autophagic flux, mock- and EBV-infected HCoEpC, were incubated with an inhibitor of the lysosomal V-APTase, Bafilomycin A1 (20 nM, Baf, Sigma-Aldrich, 88,899–55–2), for the last 4 h 3 days post-infection (p.i.),.

To inhibit proteasome, mock- and EBV-infected HCoEpC were incubated with MG132 (10 μM, Selleckchem, S2619) during the last 8 h, 3 days p.i.

DMSO was used as vehicle in all the experiments and mock-infected HCoEpC were used as control.

### 2.8 Chemiluminescent immunometric assay

Interleukin-6 (IL-6), Interleukin-8 (IL-8), vascular endothelial growth factor (VEGF), CXCL13 and CCL2 amount, released in the supernatants of mock- or EBV-infected HCoEpC pre-treated or not with PD, PAA or 5-AZA, was assessed by Magnetic Luminex assay using a human premixed multi-analyte kit (R&D Systems Bio-Techne, LXSAHM, Minneapolis, MN, USA).

### 2.9 Trans-well migration and B lymphocytes infection and proliferation

To evaluate B lymphocytes recruitment by chemokines released by mock- and EBV-infected HCoEpC, 2 × 10^6^ B lymphocytes were seeded onto 8 μm pore size transwell (Thermoscientific, CC insert MD24 8MY), in the presence of 500 μl of mock- or EBV-infected HCoEpC supernatants and cultured for 16 h at 37 °C. Then, cell counts were performed using a Neubauer chamber and a trypan blue staining. qPCR was carried out on trans-migrated B lymphocytes to assess if they were EBV-infected, using ELITE MGB kit (ELITech) as above described.

B lymphocytes were further cultured and counted with Neubauer chamber after trypan blue staining at different time point after the trans-well migration assay.

### 2.10 Western blotting

Mock- and EBV-infected HCoEpC were washed twice in PBS, pelleted and lysed in RIPA buffer (NaCl 150 mM, NP40 1%, Tris–HCl pH8 50 mM, deoxycholic acid 0.5%, SDS 0.1%) containing protease and phosphatase inhibitors. 10 µg of proteins or 30 µg for EBNA1 detection were loaded on a precast polyacrylamide gel (Bolt™ 4–12% Bis-Tris Plus, Invitrogen, Waltham, MA, USA) and then transferred to a nitrocellulose membrane (Amersham ™), that was subsequently incubated for 30 min in a blocking solution (PBS, 0.1% Tween 20, 2% BSA). Next, membranes were incubated with primary antibody for 1 h at room temperature or overnight at 4 °C. After three washing in PBS-0.1% Tween 20 (washing solution), membranes were incubated for 30 min with a secondary antibody conjugated to horseradish peroxidase. Finally, membranes were washed three times with washing solution and protein detection as well as densitometric analysis were performed through a chemiluminescence kit Western Bright ECL (Advansta, Menio Park, CA, USA) and using ImageJ software, respectively.

### 2.11 Antibodies

The following antibodies were used: mouse monoclonal anti-phospho ERK1/2 (Santa Cruz Biotechnology Inc., Biotechnology Inc., Cat# sc-7383, Dallas, TX, USA) 1:500; rabbit polyclonal anti-ERK1 and anti-ERK2 (Santa Cruz Biotechnology Inc., Biotechnology Inc., Cat# sc-93 and Cat# sc-154, Dallas, TX, USA) 1:500; mouse monoclonal anti-EBNA-1 (1EB12) (Santa Cruz Biotechnology Inc., Cat# sc-81,581, Dallas, TX, USA) 1:100; rabbit polyclonal anti-gp 350/220 (SinoBiological, Cat#40,373-T62) 1:500; mouse monoclonal anti-CHK1 (G-4) (Santa Cruz Biotechnology Inc., Cat# sc-8408, Dallas, TX, USA) 1:100; rabbit polyclonal anti- Phospho-CHK1 (Ser345) (133D3) (Cell Signaling, Cat# 2348, Danvers, Massachusetts, USA) 1:250; mouse monoclonal anti-phosphoH2AX (Santa Cruz Biotechnology Inc., Cat# sc-517,348, Dallas, TX, USA) 1:100; rabbit polyclonal anti-RAD51 (D4B10) (Cell Signaling Cat# 8875, Danvers, Massachusetts, USA) 1:500; rabbit polyclonal anti-LC3 I/II (Novus, Cat# NB100–2220, Centennial, CO, USA); rabbit polyclonal anti-SQSTM1/p62 (Novus, Cat# NBP-48,320, Centennial, CO, USA) 1:4000; rabbit polyclonal anti-DNMT1 (Proteintech, Cat #24,206–1-AP) 1:500; mouse monoclonal anti-β Actin (Sigma Aldrich, Cat# A5441, Burlington, MA, USA) 1:10,000.

Goat polyclonal anti-mouse 1:15,000 (Santa Cruz Biotechnology Inc., Cat# sc-2005, Dallas, TX, USA) and anti-rabbit 1:15,000 (Santa Cruz Biotechnology Inc., Cat# sc-2004, Dallas, TX, USA) were used as secondary antibodies.

### 2.12 Densitometric analysis

The quantification of protein bands was performed by densitometric analysis using the Image J software (1.47 version, NIH, Bethesda, MD, USA), which was downloaded from the NIH website (http://imagej.nih.gov, accessed on 10 February 2022).

### 2.13 Statistical analysis

Results are shown as the mean ± standard deviation (SD) of three independent experiments. Statistical analysis was performed with Graphpad Prism® software (Graphpad software Inc., La Jolla, CA, USA) and Bonferrroni test (as multiple comparisons test) was used to measure statistical significance. Difference was considered as statistically significant when p-value was: * < 0.05; ** < 0.01; *** < 0.001 and **** < 0.0001. When p-value was ≥ 0.05 was considered not significant (ns).

## 3 Results

### 3.1 EBV infects HCOEPC

**Fig. 1:**
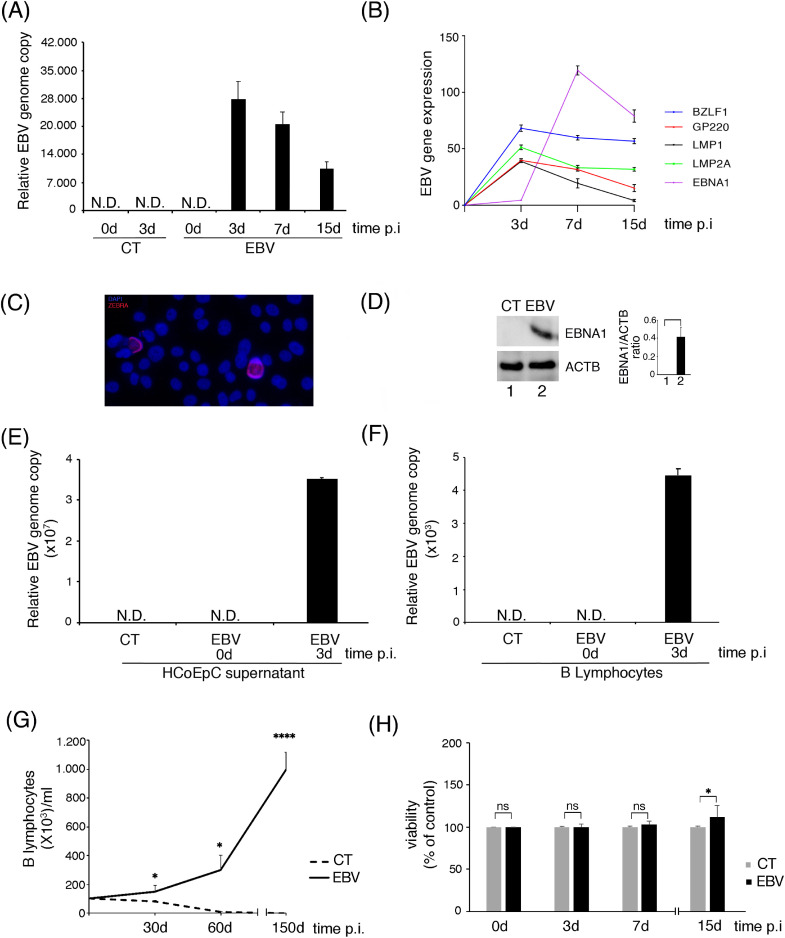
**EBV infects HCoEpC and leads to production of viral progeny able to infect primary B lymphocytes**. (A) qPCR on DNA extracted from EBV and mock-infected (CT) HCoEpC at different time point post-infection (p.i.). Relative EBV genome copy is shown. (B) qRT-PCR to analyze EBV lytic (BZLF1 and gp220) and latent (LMP2A, LMP1 and EBNA1) gene expression at different time points p.i. (C) IFA on EBV-infected HCoEpC stained with anti-ZEBRA monoclonal antibody at 15 days p.i. Nuclei were stained with DAPI (blue) (D) Western blot analysis showing EBNA1 expression in EBV and mock-infected (CT) HCoEpC at 15 days p.i. The histograms represent the mean plus SD of the densitometric analysis of the ratio of EBNA1/ACTB of three different experiments. (E) qPCR on supernatant of mock- and EBV-infected HCoEpC at 0 and 3 days p.i., to evaluate whether the infection was productive. Relative EBV genome copy is shown. (F) qPCR on DNA extracted from primary B lymphocytes exposed to supernatants of mock- and EBV-infected HCoEpC at 0 and 3 days p.i. Relative EBV genome copy is shown. (G) EBV- and mock-infected B lymphocyte proliferation evaluated at different time points p.i. (H) EBV- and mock-infected HCoEpC cell viability and proliferation were determined by MTT at different time points p.i.

We first assessed if EBV could infect HCoEpC, by evaluating the presence of viral DNA by qPCR at different time points post infection (p.i). As shown in Fig. 1A, viral DNA was detected in HCoEpC from 3 to 15 days post-infection. We then assayed the expression of a panel of latent and lytic viral genes at the same time points and found that LMP1, LMP2A, BZLF1 and gp220 were highly expressed at 3 days p.i. while EBNA1 expression increased after 7 days. Furthermore, the expression of LMP1 and gp220 genes dropped down between 7 and 15 days while EBNA1, LMP2A and BZLF1 continued to be expressed (Fig. 1B). No signal for the analyzed EBV genes was detected by RT-qPCR in the absence of reverse-transcriptase (data not shown). The expression of ZEBRA (Fig. 1C) and EBNA1 (Fig. 1D) was also demonstrated at protein level after 15 days of EBV infection. Next, to evaluate whether EBV-infected HCoEpC could release viral particles, we measured viral DNA by qPCR in cell supernatant at day 3 post EBV infection, when gp350/220 late lytic antigen was expressed. We found that EBV DNA was present in the supernatant, suggesting a productive infection (Fig. 1E). Of note, the virus released by HCoEpC was able to infect primary B lymphocytes Fig. 1F), strongly increasing their proliferation rate (Fig. 1G).

The impact of infection on HCoEpC proliferation was also monitored up to 30 days and, as shown in Fig. 1H, a slightly increase of cell proliferation was observed in EBV-infected cells compared to the mock-infected control cells.

### 3.2 Viral lytic antigens contribute to the release of inflammatory cytokines and chemokines able to recruit B lymphocytes

**Fig. 2:**
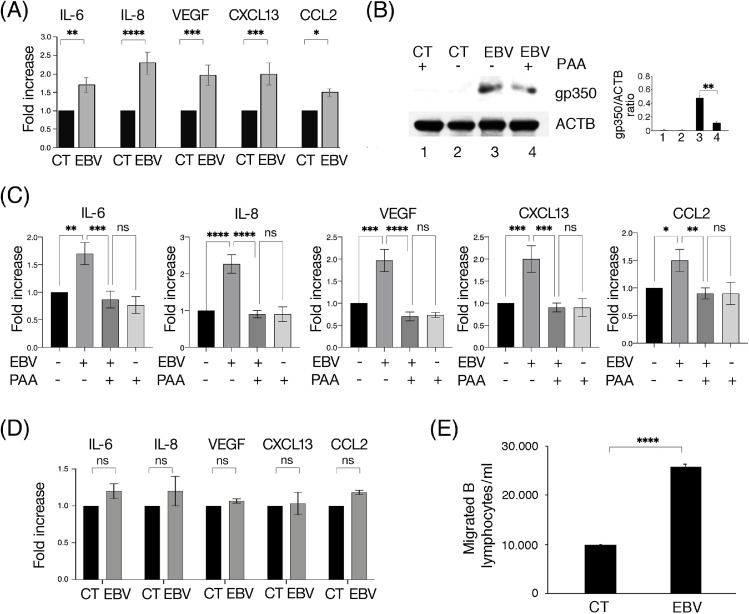
**Inflammatory cytokines and chemokines are released during EBV lytic cycle by infected HCoEpC and attract primary B lymphocytes**. (A) Magnetic Luminex assay performed to detect IL-6, IL-8, VEGF, CXCL13 and CCL2 in supernatants of mock- (CT) and EBV-infected (EBV) HCoEpC at 3 days p.i. (B) Gp350/220 (gp350) EBV late lytic gene expression, in the presence or not of Phosphonacetic acid (500 μM, PAA), was analyzed by Western blotting 3 days p.i. The histograms represent the mean plus SD of the densitometric analysis of the ratio of Gp350–220 /ACTB of three different experiments. (C) Detection of IL-6, IL-8, VEGF, CXCL13 and CCL2 by magnetic Luminex assay at 3 days p.i. in supernatants of mock- and EBV-infected HCoEpC, in the presence or not of 500 μM PAA. (D) IL-6, IL-8, VEGF, CXCL13 and CCL2 in supernatants of mock- and EBV-infected HCoEpC assessed by magnetic Luminex assay performed at 15 days p.i. (E) Transwell migration assay to evaluate the number of primary B lymphocytes recruited by supernatants of mock- and EBV-infected HCoEpC at 3 days p.i.

We then evaluated whether EBV could alter the production of cytokines by HCoEpC-infected. As shown in Fig. 2A, the release of pro-inflammatory and angiogenic cytokines, such as interleukin (IL)−6, vascular endothelial growth factor (VEGF) and chemokines such as IL-8, C-X-C motif Chemokine ligand 13 (CXCL13) and C—C Motif Chemokine Ligand 2 (CCL2), increased in EBV-infected cells compared to control cells, 3 days post-infection. Since a lytic infection occurred at this time point in HCoEpC, we investigated the role of EBV late lytic antigens in cytokine/chemokine release by treating cells with the herpesvirus DNA replication inhibitor Phosphonacetic acid (PAA). This drug that slightly the cell viability (Supplementary Fig. S1), inhibited the expression of the late EBV antigen gp350/220 (Fig. 2B) and strongly reduced the production of cytokines and chemokines (Fig. 2C), suggesting that the lytic infection was sustaining their production.

To further assess the role of viral proteins on the release of cytokines by HCoEpC-infected cells, we performed a chemiluminescent immunometric assay 15 days after infection, when the expression of late lytic protein was not-longer detected. As shown in Fig. 2D, the production of cytokines and chemokines by infected cells strongly decreased compared to that observed at 3 days post-infection, further highlighting the role of the late lytic antigens in their release. As EBV infection of HCoEpC increased the release of CXCL13 by HCoEpC, one of the most important chemokines attracting B lymphocytes, we performed an overnight migration assay using mock- or EBV-infected HCoEpC supernatants to attract B lymphocytes, in trans-well-plates. We found that the supernatant of EBV-infected cells attracted a greater number of B lymphocytes compared to the mock-infected HCoEpC (Fig. 2E). The high number of B cells recruited by infected HCoEpC and their infection by the virus released by HCoEpC (Fig. 1D) could exacerbate the inflammatory process and might correlate with the increased risk of EBV-positive lymphomas in IBD patients (Dayharsh et al., 2002).

### 3.3 EBV lytic infection activates ERK1/2 in HCOEPC promoting cytokine release

**Fig. 3:**
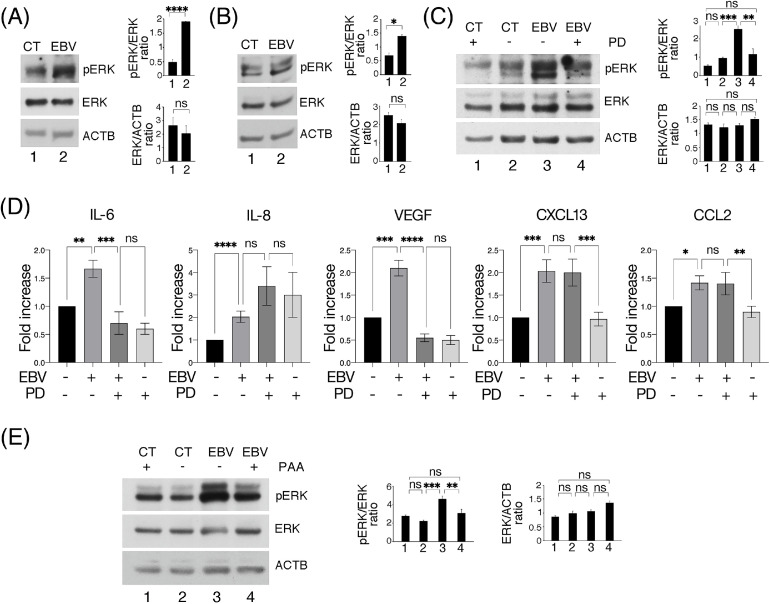
**EBV lytic infection induces ERK1/2 activation that contributes to cytokines release by HCoEpC**. (A) ERK1/2 activation (pERK) in mock- (CT) and EBV-infected (EBV) HCoEpC was assessed by Western blotting at 3 days p.i. (B) ERK1/2 activation (pERK) in mock- and EBV-infected HCoEpC at 15 days p.i.. (C) ERK1/2 activation (pERK) in mock- and EBV-infected HCoEpC at 3 days p.i., in the presence or not of ERK1/2 inhibitor PD0325901 (1 μM, PD). (D) Magnetic Luminex assay to detect IL-6, IL-8, VEGF, CXCL13 and CCL2 in supernatants of mock- and EBV-infected HCoEpC, in the presence or not of PD0325901 (1 μM, PD), at 3 days p.i. (E) Western blotting analysis to evaluate pERK level in mock- and EBV-infected HCoEpC, in the presence or not of PAA (500 μM) at 3 days p.i.. In the figure, histograms represent the mean plus SD of the densitometric analysis of the ratio of pERK/ERK and ERK/ACTB of three different experiments.

Among pro-inflammatory pathways, ERK1/2 has been reported to be activated by EBV (Luo et al., 2021). Therefore, we evaluated ERK1/2 phosphorylation in HCoEpC and found that it strongly increased after 3 days of EBV infection (Fig. 3A) while decreased 15 days post-infection (Fig. 3B). These results mirrored the release of pro-inflammatory cytokines observed after 3 and 15 days of infection (Fig. 2A and 2D) and interestingly, a positive feed-back loop may occur between inflammatory cytokines and ERK1/2 (D'Orazi et al., 2021). In order to assess the role of ERK1/2 in pro-inflammatory cytokine secretion, we inhibited it by using PD 0,325,901 (PD) (Fig. 3C) and found that IL-6 and VEGF cytokine release by EBV-infected HCoEpC strongly decreased (Fig. 3D), while cell viability was slightly influenced by this drug (Supplementary Fig. S1). This suggests that ERK1/2 may contribute to promote virus-induced inflammation. We then found that the inhibition of viral replication by PAA, besides reducing cytokine release, counteracted ERK1/2 phosphorylation (Fig. 3E), indicating that the EBV late lytic antigens were involved also in this effect.

### 3.4 ERK1/2 activation contributes to the impairment of DDR in EBV-infected HCOEPC

**Fig. 4:**
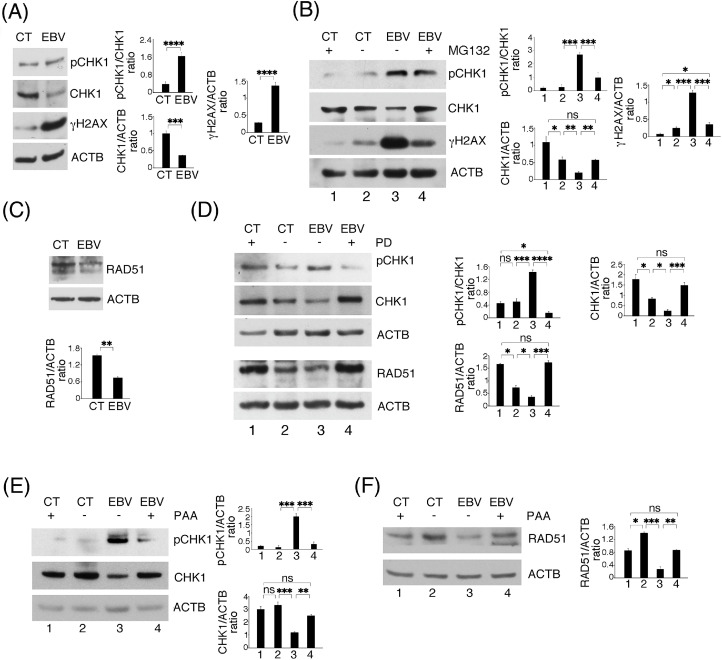
**EBV lytic cycle dysregulates DDR and drives CHK1 to proteasome degradation by activating ERK1/2 in HCoEpC**. (A) Western blotting performed to analyze phospho-CHK1 (pCHK1), CHK1 and phospho-H2AX (γH2AX) levels in mock- (CT) and EBV-infected (EBV) HCoEpC. (B) Amount of pCHK1, CHK1 and γH2AX in mock- and EBV-infected HCoEpC, in the presence or not of proteasome inhibitor MG132 (10 μM). (C) RAD51 in mock- and EBV-infected HCoEpC. (D) pCHK1, CHK1 and RAD51 in mock- and EBV-infected HCoEpC, in the presence or not of PD PD0325901 (1 μM, PD), detected by Western blotting. (E) pCHK1, CHK1 and F) RAD51 expression in mock- and EBV-infected HCoEpC, in the presence or not of PAA (500 μM). In the figure, histograms represent the mean plus SD of the densitometric analysis of the ratio of pCHK1/CHK1, CHK1/ACTB, γH2AX/ ACTB and RAD51/ACTB at 3 days p.i, of three different experiments.

Inflammation may be linked to carcinogenesis also by favoring genomic instability. This process is tightly controlled by DDR that plays a critical role in preserving genome stability and preventing tumorigenesis. Among the DDR molecules, there is checkpoint kinase 1 (CHK1), which induces cell cycle arrest, allowing DNA damage repair (Neizer-Ashun et al., 2021). Interestingly, the phosphorylation of CHK1, mainly mediated by ATR, besides activating it, marks this protein for proteolytic degradation (Zhang et al., 2005). Notably, protein phosphatase 2A (PP2A), mediating CHK1 de-phosphorylation, can be inhibited by ERK1/2 (Liu et al., 2019). Here we asked if ERK1/2, activated by EBV, could promote both CHK1 phosphorylation and degradation and impair DDR in HCoEpC-infected cells. As shown in Fig. 4A, we found that in EBV-infected HCoEpC CHK1 was hyper-phosphorylated and the expression of total molecule was reduced. These effects correlated with a higher DNA damage, as evidenced by the increased phosphorylation of H2AX (γH2AX) (Fig. 4A). To confirm that CHK1 degradation occurred via proteasome, we used their inhibitor MG132. This drug, whitout influencing cell viability (Supplemetary Fig. S1), rescued CHK1expression and, accordingly, resulted in a reduced H2AX phosphorylation in EBV-infected HCoEpC (Fig. 4B). CHK1 is a key regulator of genome integrity maintenance and its activity is known to be required for that of RAD51, a molecule belonging to the homologous repair (HR) of DNA damage (Sorensen et al., 2005). Interestingly, also the expression of RAD51 was downregulated by EBV in HCoEpC (Fig. 4C), which may further induce genome instability. Next, we found that the ERK1/2 inhibitor PD reverted the hyperphosphorylation of CHK1and its reduction and counteracted RAD51 downregulation as well in EBV-infected cells (Fig. 4D), suggesting that ERK1/2 activation was strongly involved in the impairment of DDR. As the inhibition of EBV lytic cycle by PAA counteracted ERK1/2 activation (Fig. 3E), we asked whether PAA could also lead to CHK1 de-phosphorylation and rescue its expression level and that of RAD51, downregulated by EBV. As shown in Fig. 4E and 4F, the phosphorylation of CHK1 was reduced and the expression of both proteins was restored by PAA in EBV-infected HCoEpC, similarly to what observed with PD, reinforcing the link between EBV late lytic antigen expression, ERK1/2 activation and DDR dysregulation.

### 3.5 EBV-mediated ERK1/2 activation reduces autophagy in HCOEPC

**Fig. 5:**
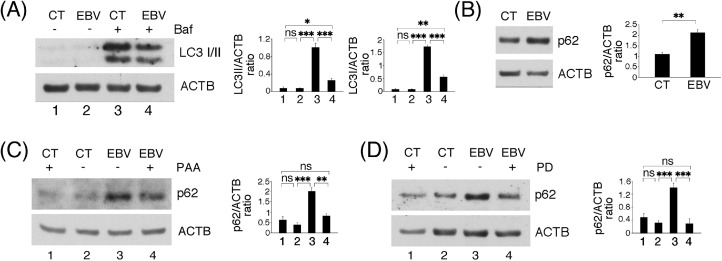
**ERK1/2 activation contributes to impairment of autophagy in lytically infected HCoEpC**. (A) Western blotting performed to analyze LC3II level in mock- (CT) and EBV-infected (EBV) HCoEpC 3 days p.i., treated or not with Bafilomycin A1 (20 nM) for the last 4 h. (B) SQSTM1/p62 (p62) expression in mock- and EBV-infected HCoEpC revealed by western blotting. (C) p62 level detected by Western blotting in mock- and EBV-infected HCoEpC, in the presence or not of PAA (500 μM). (D) p62 expression level in mock- and EBV-infected HCoEpC in the presence or not of PD0325901 (1 μM, PD). Histograms represent the mean plus SD of the densitometric analysis of the ratio of LC3II/ACTB, LC3I/ACTB and p62/ACTB at 3 days p.i., of three different experiments.

We have previously reported that EBV replication can block the autophagic flux in lymphoma cells (Granato et al., 2014). Here we asked whether EBV replication in HCoEpC could affect autophagy, as autophagy plays an important role in the prevention of cancer onset (Cirone et al., 2019). As shown in Fig. 5A and 5B, EBV reduced LC3II and induced the accumulation of p62/SQSTM1, both autophagic markers, suggesting a reduction of this process. Notably, the inhibition of the viral lytic cycle by PPA or the use of ERK1/2 inhibitor PD counteracted the impairment of autophagy, as suggested by the reduced expression level of autophagic marker p62/SQSTM1 (Fig. 5C and 5D). These findings suggest that the EBV late lytic antigens and the activation of ERK1/2 were impairing autophagy in EBV-infected HCoEpC, effect that, together with inflammation and DDR dysregulation, could favor oncogenesis.

### 3.6 The demethylating agent 5-AZA counteracts ERK1/2 activation and DDR and autophagy dysregulation induced by EBV

**Fig. 6:**
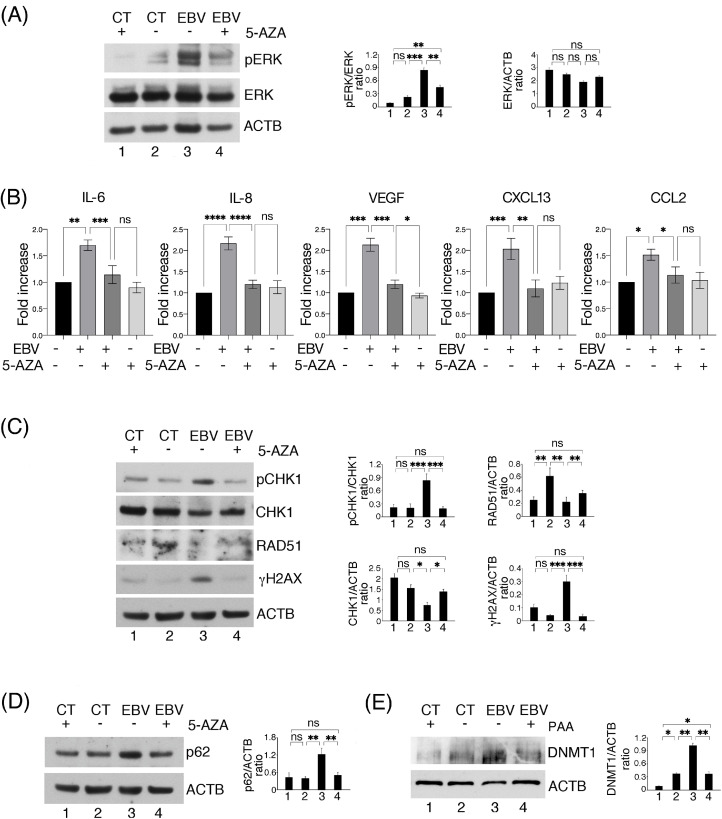
**DNA methylation correlates with ERK1/2 activation and DDR and autophagy impairment in EBV-infected HCoEpC**. (A) ERK1/2 activation (pERK) in mock- (CT) and EBV-infected (EBV) HCoEpC, in the presence or not of -Azacytidine (0.5 μM, 5-Aza), assessed by Western blotting. (B) Magnetic Luminex assay to detect IL-6, IL-8, VEGF, CXCL13and CCL2 in supernatants of mock- and EBV-infected HCoEpC, in the presence or not of 5-Aza (0.5 μM). (C) pCHK1, CHK1, γH2AX and RAD51 level in mock- and EBV-infected HCoEpC, in the presence or not of 5-Aza (0.5 μM). (D) p62 expression in mock- and EBV-infected HCoEpC, in the presence or not of 5-Aza (0.5 μM). (E) DNMT1 level in EBV-infected HCoEpC at 3 days p.i., in the presence or not of PAA (500 μM). Histograms represent the mean plus SD of the densitometric analysis of the ratio of pCHK1/CHK1, CHK1/ACTB, γH2AX/ACTB, RAD51/ACTB, p62/ACTB and DNMT1/ACTB at 3 days p.i, of three different experiments.

DNA methylation, catalyzed by DNA methyltransferase enzymes (DNMTs), is one of the most important epigenetic modifications involved in carcinogenesis (Locke et al., 2019). As for other oncoviruses, EBV infection is known to promote this epigenetic change in several cell types (Leong and Lung, 2021). Here we found that 5-AZA de-methylating agent was able to counteract EBV-induced ERK1/2 activation (Fig. 6A), cytokine production (Fig. 6B), phospho- and total CHK1 and RAD51 dysregulation and prevent DNA damage induction (Fig. 6C). Finally, p62/SQSTM1 accumulation was also reduced by 5-AZA in EBV-infected HCoEpC (Fig. 6D), suggesting that hypermethylation contributed also to autophagy inhibition. All together these findings suggest that EBV infection promoted DNA hypermethylation that contributed to ERK1/2 activation, inflammatory cytokine release and DDR and autophagy dysregulation in HCoEpC. Moreover, we found that EBV increased DNA methyltransferase 1 (DNMT1) expression in the infected cells (Fig. 6E), which was reduced by PAA, indicating that the viral lytic infection was upregulating it and that this epigenetic change was promoting inflammation and other effects predisposing to carcinogenesis.

## 4 Discussion

EBV has been associated with more than 79% of cases of IBD, in particular with the most severe and refractory forms of these diseases (Xu et al., 2020; Ciccocioppo et al., 2016) that may increase the risk of colon carcinogenesis (Axelrad et al., 2016). The association of EBV with human cancers, including NPC and gastric carcinomas, has been clearly demonstrated (Delecluse et al., 2007), but whether or not the virus might act as cofactor in promoting the onset of colon cancer and whether it can occur through increasing inflammation and favoring IBD, remains an open question. In this study we show for the first time that EBV infects HCoEpC, increases the release of several pro-inflammatory cytokines and alters cellular processes playing a key role in maintaining genome integrity and proteostasis, such as DDR autophagy. We found that the production of pro-inflammatory mediators and the above-mentioned effects correlated with the lytic phase of EBV infection in HCoEpC. Despite the production of viral particles, these cells did not undergo cell death but their proliferation rate rather increased compared to mock-infected cells. A chemokine attracting B lymphocytes, namely CXCL13, was also produced by EBV-infected HCoEpC. B lymphocytes recruited by this chemokine became themselves infected by EBV, which could exacerbate inflammation. If the release of CXCL13 could occur *in vivo*, it could promote the onset of B cell lymphomas, malignancies that indeed more frequently arise in patients suffering of IBD (Nissen et al., 2015). Although latent antigens, such as LMP1 and LMP2A, have been shown to play a key role in EBV-driven cancers, including NPC and GC (Leong and Lung, 2021), the contribution of lytic phase to viral-driven carcinogenesis is now emerging, based on several evidences (Manners et al., 2018). Among those, the reduced capacity to drive lymphomagenesis in SCID mice by EBV mutants that cannot undergo viral replication (Hong et al., 2005) and the activation of pro-inflammatory/pro-tumorigenic pathways occurring during the lytic phase of viral life cycle that may increase the risk of cancer onset (Rosemarie and Sugden, 2020). Accordingly, in this study, the EBV lytic infection correlated with pro-inflammatory cytokine release and the activation of ERK1/2. This pathway, often phosphorylated in IBD (Broom et al., 2009), is considered to be strongly involved in both inflammation and carcinogenesis (Birkner et al., 2017; Montagut and Settleman, 2009). Importantly, here we showed that ERK1/2 phosphorylation contributed to the impairment of DDR in HCoEpC infected by EBV as, its activation increased the phosphorylation and the proteasomal degradation of CHK1 and reduced the expression level of RAD51 as well. These molecules play a key role in DDR, particularly in the HR pathway, that is the most important error free DNA repair able to preserve DNA integrity (Qing et al., 2021). The downregulation of these molecules indeed resulted in an increased DNA damage in EBV-infected HCoEpC. These findings may have important implications in carcinogenesis, as DNA damage and the failure to repair it represent key risk factors for the development of cancer (Alhmoud et al., 2020). Previous studies have reported that EBV could alter DDR during the lytic phase of cell cycle in other cell types (Hau and Tsao, 2017), effect to which contributed ZEBRA (Bailey et al., 2009) and BGLF4 tegument protein (Ho et al., 2018).

A critical role in preventing oncogenic transformation is also played by the autophagic process, mainly through the degradation of damaged organelles producing ROS such as mitochondria (Cirone et al., 2019). Here, we found that EBV dysregulated also this process in HCoEpC, leading to the accumulation of p62/SQSTM1. This protein has been reported to bridge autophagy with DDR given that its increased expression, due to autophagy inhibition, can promote the degradation of DDR key molecules (Hewitt et al., 2016) as observed also in EBV-immortalized LCLs (Wang et al., 2019). However, the role of p62/SQSTM1 in the first steps of EBV-carcinogenesis is quite controversial, since we recently found that p62/SQTSM1 accumulation stabilized NRF2, reduced ROS and inflammatory cytokine release, counteracting the process of viral-driven B cell *in-vitro* immortalization (Gilardini Montani et al., 2022).

We have also previously shown that EBV blocked autophagy during the activation of the lytic cycle to facilitate the intracellular transportation of viral particles (Granato et al., 2014). Although EBV has been shown to induce DNA hypermethylation (Zhang et al., 2022) and DNA and histone methylation have been reported to put a brake on autophagy (Shi et al., 2021) and impair DDR (Gong and Miller, 2019), the present study evidenced for the first time that, through this epigenetic modification, EBV dysregulated DDR and autophagy in HCoEpC. Indeed, we found that the DNA-demethylating agent 5-AZA counteracted autophagy and DDR dysregulation induced by the virus and restrained cytokine release and ERK1/2 activation as well. These findings, together with the increased expression of DMT1 induced by EBV infection, suggest that methylation may represent a key event in EBV-driven inflammation and other pro-tumorigenic effects and indicate that, in future studies, it will be important to explore the entire methylome in EBV-and mock-infected HCoEpC. It has been shown that methylation occurs in cervical lesions and cancer associated with HPV and methylation levels may correlate with disease progression (Clarke et al., 2018). Moreover, Helicobacter Pylori, that like EBV can promote inflammation-driven epithelial carcinogenesis, can also increase DNA methylation to repress the expression of genes involved in the control of autophagy, DDR and to promote inflammation (Muhammad et al., 2017). This suggests that, as in the case of EBV, methylation is a common mechanism exploited by oncoviruses, belonging to different families, to control both host and their own gene expression, in order to promote malignancies (Pietropaolo et al., 2021).

Discovering new therapeutic strategies able to interfere with methylation, could lead to important advances in the prevention of virus-associated pathologies including cancer and possibly also help to reduce viral-mediated immune suppression.

## Author contributions

R.S. investigation, visualization, methodology, data curation, formal analysis, validation, writing-review and editing; L.E., C.P., S.L.P., A.R.: investigation, visualization, methodology, formal analysis. Software. A.A.; methodology, validation; A.G. investigation; R.G. validation; M.S.G.M.: data curation, formal analysis, validation, writing—review and editing; M.C.: conceptualization, resources, data curation, formal analysis, funding acquisition, validation, project administration, writing-review and editing. All authors have read and agreed to the published version of the manuscript.

## Ethics approval and consent to participate

This research involving human subjects has been performed in accordance with the Declaration of Helsinki and has been approved by the ethic committee of Policlinico Umberto I, Rome, Italy (847/19).

## Funding informations

This work was supported by the 10.13039/501100005010Italian Association for Cancer Research (AIRC; grant IG 2019 Id.23040), PRIN2017 (2017K55HLC) and by ATENEO 2019.

## Declaration of Competing Interest

The authors declare that they have no known competing financial interests or personal relationships that could have appeared to influence the work reported in this paper.

## Data Availability

Data will be made available on request.

## Appendix Supplementary materials

Image, image 1
