# Supplementary figures and images for: EBV infection of primary colonic epithelial cells causes inflammation, DDR and autophagy dysregulation, effects that may predispose to IBD and carcinogenesis
Source: Virus Res. 2023 Oct 12;338:199236. doi: 10.1016/j.virusres.2023.199236 (PMC10582763; doi:10.1016/j.virusres.2023.199236)

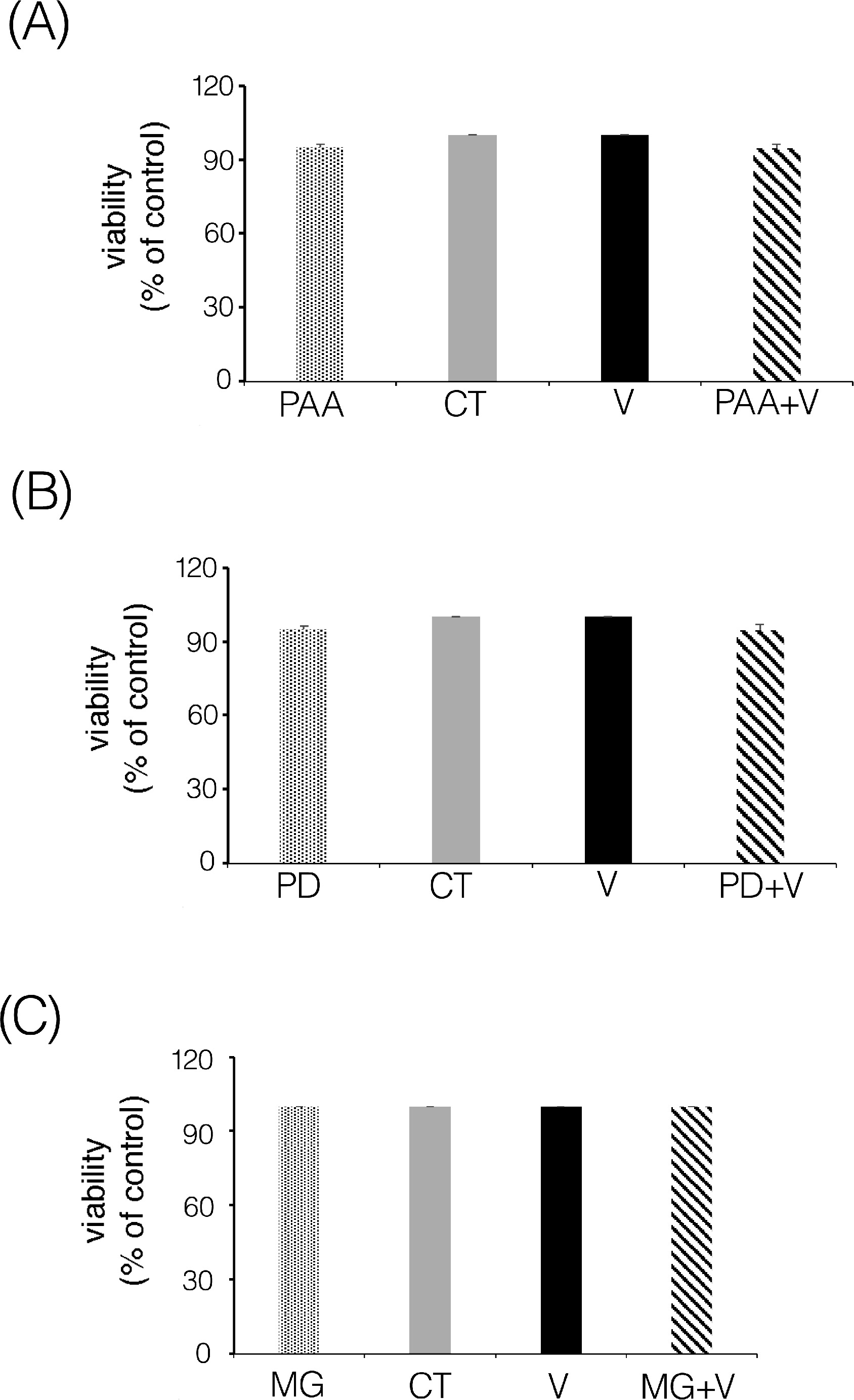

Supplement: Supplementary file 1 [file mmc1.jpg]
